# Non-contact method for directing electrotaxis

**DOI:** 10.1038/srep11005

**Published:** 2015-06-09

**Authors:** Dinesh K. Ahirwar, Mohd W. Nasser, Travis H. Jones, Emily K. Sequin, Joseph D. West, Timothy L. Henthorne, Joshua Javor, Aniruddha M. Kaushik, Ramesh K. Ganju, Vish V. Subramaniam

**Affiliations:** 1 Department of Pathology, College of Medicine, The Ohio State University, Columbus, Ohio, U.S.A; 2 Department of Mechanical & Aerospace Engineering, The Ohio State University, Columbus, Ohio, U.S.A; 3 Department of Chemistry and Biochemistry, The Ohio State University, Columbus, Ohio, U.S.A

## Abstract

We present a method to induce electric fields and drive electrotaxis (galvanotaxis) without the need for electrodes to be in contact with the media containing the cell cultures. We report experimental results using a modification of the transmembrane assay, demonstrating the hindrance of migration of breast cancer cells (SCP2) when an induced a.c. electric field is present in the appropriate direction (i.e. in the direction of migration). Of significance is that migration of these cells is hindered at electric field strengths many orders of magnitude (5 to 6) below those previously reported for d.c. electrotaxis, and even in the presence of a chemokine (SDF-1α) or a growth factor (EGF). Induced a.c. electric fields applied in the direction of migration are also shown to hinder motility of non-transformed human mammary epithelial cells (MCF10A) in the presence of the growth factor EGF. In addition, we also show how our method can be applied to other cell migration assays (scratch assay), and by changing the coil design and holder, that it is also compatible with commercially available multi-well culture plates.

Cell migration is important in several physiologically relevant situations such as embryonic development, wound healing, and metastasis of cancer. Non-ciliated cells migrate in response to gradients in chemical composition (chemotaxis), mechanical forces, and electric fields (galvanotaxis or electrotaxis). The latter has been observed for over a hundred years since the report of Dineur in 1892^1^, where the author proposed the use of the term galvanotaxis to describe migration of leukocytes in the presence of a d.c. electric field:

“Je crois pouvoir conclure de ces experiences, qu’il existe chez le leucocyte une sensibilite speciale a l’electricite. Je propose de lui donner le nom de <<galvanotaxisme>>.” (Translated: I believe I can conclude from these experiments, that there exists among the leucocytes a special sensitivity to electricity. I propose to give it the name “galvanotaxism”.)

Since Dineur’s report, many vertebrate cells have been observed to exhibit galvanotaxis^2^ or electrotaxis as it is now called. The majority of *in vitro* electrotaxis experiments are conducted under the action of a d.c. field, and involve metal electrodes directly inserted into the medium containing the cells or in indirect contact through agar or salt bridges^3^,^4^,^5^,^6^,^7^,^8^. The threshold for cells to sense an electric field *in vitro* has been reported^9^ to be >10 mV/cm, with magnitudes of d.c. electric fields on the order of 0.1–10 V/cm required for observing electrotaxis^9^,^10^. At these electric field strengths, effects of localized heating can be non-negligible^10^.

Recently, electrotaxis experiments in a.c. fields of very low frequencies^3^ on the order of mHz, and a.c. fields from 1.6 Hz to 160 Hz applied together with d.c. fields have been reported^11^. These experiments show that collective cell migration^3^, direction of cell migration and migration speed can all be controlled by application of electric fields. Despite the use of modern patterning techniques for shaping d.c. electric fields and use of microfluidic devices^3^,^4^,^5^,^6^,^7^,^8^, the methods of applying these d.c. and very low-frequency a.c. electric fields still involve either direct contact or indirect contact (via agar bridges) with the media containing the cells. Since the methods of applying electric fields have changed little over the past several decades^3^, there is a need for new electrotaxis assays^12^ and methods of applying electric fields in a non-contact manner.

In this article we describe how a well-known assay for chemotaxis referred to as the transmembrane or Transwell assay^13^,^14^ may be modified to conduct non-contact electrotaxis experiments. The transmembrane assay was first described by Boyden^15^ to analyze the chemotactic response of leukocytes and is sometimes referred to as the Boyden chamber assay. This assay consists of an insert at the bottom of which is a membrane of selectable pore size (0.4 μm–12 μm), depending on the size of the cells. The insert is then placed into a well, forming two distinct compartments separated by the membrane. Cells are seeded on the top side of the membrane, and the bottom compartment may contain a chemotactic agent. The cells migrate from the top surface of the membrane through to the bottom surface. After a suitable incubation time (dependent on cell type), the number of migrated cells is counted by fixing and staining, or by staining fluorescently, removing from the membrane by dissociation (e.g. by using trypsin) and using a fluorescent reader.

We modified the standard transmembrane assay to develop a new method for inducing a.c. electrotaxis in a truly non-contact manner, without the need for electrodes and agar or salt bridges to be in contact with the medium containing the cells. Moreover, this new method enables the study of electrotactic behavior alone or electrotaxis in the presence of chemotactic agents as well. The a.c. electric fields are induced in the media containing the cells using electromagnetic induction. A time-varying current driven through a custom designed coil placed with in-house fabricated glass wells (with membrane inserts) lining either side of the coil enable an electric field to be induced in the vertical direction, along the axis of migration. The time-varying current generates a time-varying magnetic field which induces an electric field in the azimuthal direction around the coil. When the glass wells containing the membrane inserts are placed on the sides of the coil, this azimuthal field is in the direction perpendicular to the membranes and along the axis of migration. Results are presented for a highly metastatic human breast cancer cell line^16^ as well as for a non-transformed human mammary epithelial cell line^17^ (MCF10A) (Supplementary Figs. S15-S16), for a single duty cycle of 100 kHz as illustrative examples. Finally, we also show how a modification of our method can induce electric fields in a non-contact manner in standard culture plate wells, enabling visualization of actin filaments using phalloidin-fluorophore conjugates and fluorescence microscopy, and extending applicability of this method to the scratch migration assay as well.

## Results

### Weak a.c. electric fields hinder migration of highly metastatic SCP2 cells

**Figure 1:**
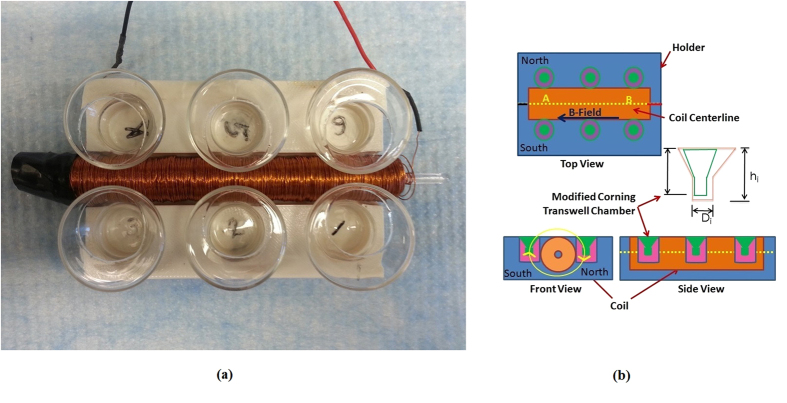
Transmembrane assay modified to incorporate an electromagnetic (EM) coil to induce electric fields to drive electrotaxis. **(a)** Photograph of modified transmembrane assay showing the custom–made glass wells, 3D printed holder apparatus, and in-house designed and fabricated EM coil. **(b)** Schematics showing the top, front, and side views of the modified transmembrane assay including EM coil and glass wells custom-made to incorporate commercially available Transwell membrane inserts. Also shown in the front view of the apparatus is the direction of the induced electric field applied over a majority (60%) of the 10 μs period.

The experimental apparatus consists of a custom made coil, holder, glass wells that can accommodate commercially available Transwell permeable inserts (8 μm pore, 24-well, Corning-Costar, Lowell, MA), and function generator (Hewlett Packard 33120A). The detailed design of the apparatus is described elsewhere (**Methods**). The coil has a d.c. resistance of 50.45 Ω, and an inductance of 14.25 mH as measured by an LCR meter (Extech Instruments Model 380193) at 1 kHz. The coil is placed at the center of the holder with six custom made glass wells on either side (Fig. 1a, Supplementary Figs. S1-S2). The glass wells have off-centered holes within which commercially available Transwell membrane inserts can be placed at closest proximity to the coil (Fig. 1b, Supplementary Fig. S1). The wells and holder are designed and fabricated so that each membrane of the transmembrane insert is positioned exactly at the same height as the centerline of the coil (Fig. 1b). One side of the coil is arbitrarily labeled “North” for ease of reference while the other is labeled “South”, with respect to the red wire labeled “East” (Fig. 1b).

Highly metastatic breast cancer cells known as SCP2 (provided by Dr. Joan Massague at Sloan-Kettering Cancer Center, New York, NY), a single cell population derived from MDA-MB-231 that is known to metastasize to the bone^16^, were cultured in Dulbecco’s Modified Eagle Medium (DMEM; Life Technologies, Gaithersburg, MD, USA), supplemented with 10% heat inactivated fetal bovine serum (FBS), 5 U/mL penicillin, and 5 mg/mL streptomycin. The SCP2 cells were placed in the upper chamber, and both compartments contained 0.1% FBS-DMEM (Fig. 1b). The entire apparatus (holder, coil, modified transmembrane chambers with Transwell inserts and cells) was placed in a 37 °C culture incubator with humidified air containing 5% CO_2_. The leads of the coil were connected to the function generator placed outside the incubator. The cells were allowed to migrate for 8 hours, and then were fixed and stained using Hema-3 stain kit according to the manufacturer’s instructions. The number of migratory cells per membrane was then measured using light microscopy by counting the total number of cells in each of 5 contiguous images spanning radially outward (5 fields) from the coil (Supplementary Fig. S3). The counts were used to determine the normalized percentage of migration, with the number of cells migrated in the control set to 100%. In order to ensure that cell migration in our modified transmembrane assay with the Transwell insert is not statistically different from the conventional transmembrane assay, control experiments were performed (Supplementary Fig. S4).

**Figure 2:**
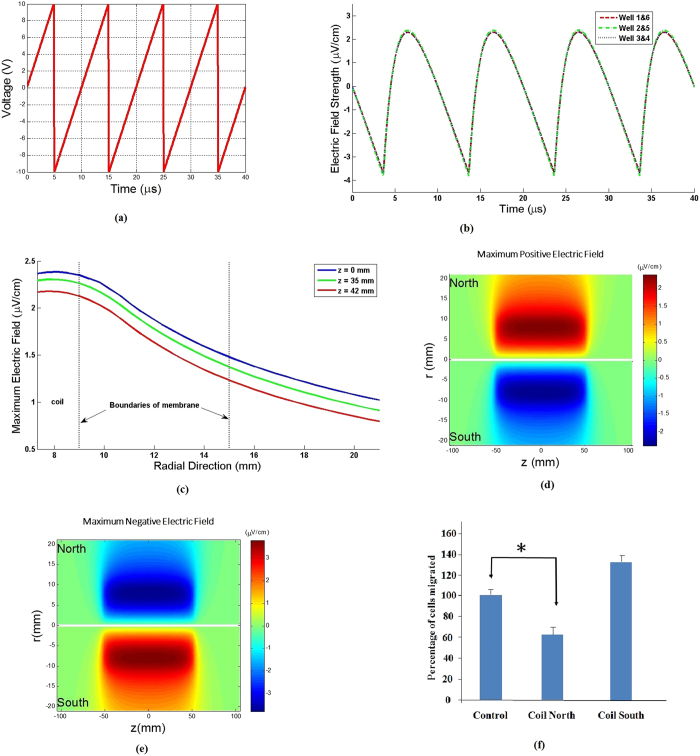
**(a)**Sawtooth shaped voltage waveform output from the function generator used to drive current through the EM coil. The sharp drop-off occurs in ~50 ns. **(b)** Time variation of the induced azimuthal electric field E_θ_ calculated from first principles (see **Methods**, Supplementary Figs.S7-S11), for all wells. This induced E field is in the vertical direction (up or down) at the membrane inserts placed on either side of the coil. Note the asymmetry over a given 10 μs interval (positive ~60% of the time and negative ~40% of the time over the interval). Based on the direction of our windings, a positive E_θ_ indicates that the induced E field is in the downward direction on the “North” side of the coil while a negative E_θ_ indicates an upwardly directed induced E field on the “North” side of the coil. **(c)** Variation of the maximum E_θ_ versus radial distance away from the coil (i.e. along the porous membrane) calculated for each axial location where the glass wells are placed. **(d)** Contour plot of the induced E field on both sides of the coil where the wells are placed, for ~60% of each 10 μs period. **(e)** Contour plot of the induced E field on both sides of the coil, for ~40% of each 10 μs period. **(f)** Results showing effects of induced E fields on migration of SCP2 cells (N = 3). Note that migration is hindered on the “North” side of the coil (p = 0.021) while it follows a weak trend of enhanced migration on the “South” side with a borderline non-significant p value (p = 0.076). The induced E field is directed downward (in the direction of migration) for ~60% per period while it is directed upward (against the direction of migration) for ~40% per period on the “North” side of the coil. These values are reversed for the “South” side of the coil.

A 20 Vpp, 100 kHz sawtooth shaped voltage waveform (Fig. 2a) was imposed on the coil, resulting in a time-varying current flow generating a time-varying magnetic field. In accordance with Faraday’s Law, the time-varying magnetic field induces an electric field. This induced electric field can be calculated (**Methods**) and is in the azimuthal direction (vertically up or down with respect to the membrane inserts). The induced electric field is asymmetric over a duty cycle (Fig. 2b), resulting in different durations of the electric field in the direction of migration on the two sides of the coil. On the side of the coil labeled “North”, the induced electric field is in the direction of migration (i.e. downward) over ~60% of a single duty cycle lasting 10 μs with a maximum magnitude of ~2.3–2.4 μV/cm (Fig. 2b-2e). Over the remaining 40% of the 10 μs period, the induced electric field on the “North” side is in the direction opposite to that of migration (i.e. upward) with a maximum magnitude of ~(−)3.7-(−)3.8 μV/cm (Fig. 2b-2e). The opposite of these trends is realized on the side labeled “South”. At any instant of time, the induced electric field decreases with increasing radial distance from the outer surface of the coil (Fig. 2c). Contours of the calculated induced electric field reveal that it is fairly uniform over the length of the coil so that each of the three wells on a given side of the coil experiences the same induced electric fields at a given instant of time (Fig. 2d and 2e). Note that the maximum induced electric fields are on the order of ~3.8 μV/cm, *at least 4 to 5 orders of magnitude smaller* than typically required for electrotaxis^9^,^10^ and *six orders of magnitude smaller* than previously reported for MDA-MB-231 cells^8^.

Experimental results with SCP2 cells indicate that migration on the “North” side of the coil is hindered (p=0.021) when compared to the control experiments where no electric field is present. However, migration on the “South” side of the coil shows a trend of increased migration which is not statistically significant (p=0.076) when compared to the controls where no electric field is present (Fig. 2f). Since the “North” side of the coil experiences an electric field in the direction of migration (i.e. downward) for a greater duration (~6 μs per period) compared to experiencing an electric field opposite to the direction of migration (~4 μs per period), it can be seen that migration of SCP2 cells is hindered when the induced electric field is in the direction of migration. This observed hindrance of migration on the “North” side is consistent with previously reported observations that MDA-MB-231 cells migrate toward the positive electrode when subjected to d.c. electric fields^8^.

### Effects of hindered migration of SCP2 cells under weak a.c. fields are reversible

**Figure 3:**
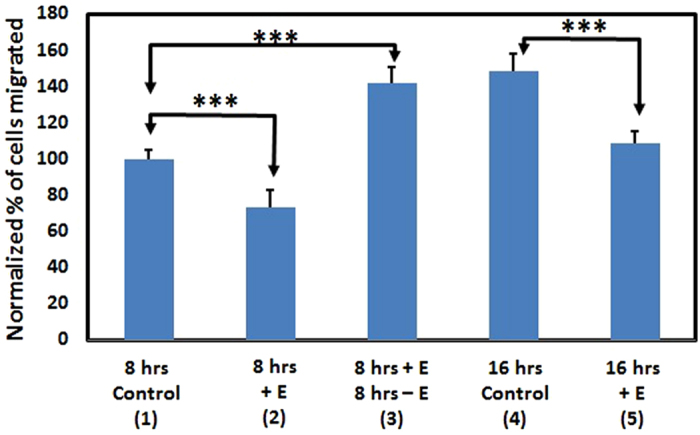
Results showing the effects of turning off the induced electric field on SCP2 cells on the “North” side of the coil after cells have been exposed to it for 8 hours. 8 hrs control (1): Control experiments used for normalization, showing cells migrated in 0.1% serum media after 8 hours with no induced E field, as 100%. **8 hrs +E (2):** Hindered migration (consistent with Fig. 2f) of SCP2 cells in the presence of an induced E field (8 hrs control (1) and 8 hrs +E (2): p < 0.001). **8 hrs +E 8 hrs −E (3):** Migration of cells after 16 hours, with the induced E field for the first eight hours and the field shut off for the next 8 hours (8 hrs control (1) and 8 hrs +E 8 hrs −E (3): p < 0.001). **16 hrs control (4):** Control experiments showing cell migration after 16 hours with no induced E field. Note that cell migration appears to be restored to normal levels despite hindered migration of SCP2 cells due to the induced E field for the first 8 hours (8 hrs +E 8 hrs −E (3) and 16 hrs control (4): p =0.342). **16 hrs +E (5):** Migration of cells after 16 hours in the presence of an induced E field (16 hrs control (4) and 16 hrs +E (5): p < 0.001)

The observed hindrance of migration of SCP2 cells under the action of weak (~1 μV/cm) induced electric fields raises the question of whether or not the ability of these cells to migrate is permanently affected after exposure to the induced electric field. In order to address this question, SCP2 cells were prepared as described before, in 0.1% serum media and control experiments were separately conducted in our modified transmembrane assay without application of the induced electric field, for 8 hours and 16 hours respectively (8 hr control (1) and 16 hrs control (4), Fig. 3). In parallel, SCP2 cells were subjected to an induced electric field generated by a sawtooth voltage waveform at 20 Vpp and 100 kHz duty cycle on the “North” side of the coil for 8 hours (8 hrs +E (2), Fig. 3, p < 0.001 for 8 hrs control (1) and 8 hrs +E (2)) and a separate set which were subjected to the electric field (“North” side) for 8 hours after which time the electric field was turned off and migration allowed to continue for another 8 hours (8 hrs +E 8 hrs −E (3), Fig. 3, p < 0.001 for 8 hrs control (1) and 8 hrs +E 8 hrs −E (3)). It can be seen from these results that as discussed before (Fig. 2f), application of the induced electric field in the direction of migration hinders SCP2 cell movement (8 hrs control (1) versus 8 hrs +E (2), Fig. 3). However, once the electric field is turned off after 8 hours, it can be seen that the SCP2 cells migrate over the next 8 hours in numbers comparable to the corresponding control case (8 hrs +E 8 hrs −E (3) versus 16 hrs control (4), Fig. 3, p=0.342 for 8 hrs +E 8 hrs −E (3) and 16 hrs control (4)). Furthermore, migration when exposed to the induced electric field for 16 hours is hindered compared to both the control (no induced electric field for 16 hours) and the case where the induced electric field is shut off after 8 hours (16 hrs +E (5) versus 8 hrs +E 8 hrs −E (3) or 16 hrs control (4), Fig. 3). These results show that the migration properties of the SCP2 cells are not irreversibly altered by our application of an induced electric field, and that the effects of electrotaxis are reversible once the induced electric field is turned off (8 hrs +E (2) and 8 hrs +E 8 hrs −E (3) versus 16 hrs control (4), Fig. 3).

### Weak a.c. electric fields hinder chemotaxis of metastatic breast cancer cells

Experiments on combined chemotaxis and electrotaxis were also performed with the SCP2 cells on the modified transmembrane assay in order to examine the effects of the induced a.c. electric field in hindering chemotaxis. Two well-known chemokines/growth factors to which SCP2 cells respond, stromal-derived factor 1-α (SDF-1α), also known as CXCL12, and epidermal growth factor (EGF), were selected for investigation and placed in the bottom compartment of the custom-made chamber of the modified transmembrane assay (Fig. 1). CXCR4 is a receptor that is overexpressed in malignant breast cancer, and is known to bind to its cognate ligand CXCL12 (SDF-1α) and has been correlated with poor prognosis^18^. EGF is known to be a growth factor that causes leading edge protrusions, an early event in migration of breast cancer cells^19^. CXCR4 positive breast cancer cells have been shown to metastasize to CXCL12 expressing organs as their first destination^20^. It has also been reported that CXCL12/CXCR4 signaling induces actin polymerization and chemotactic property of breast cancer cells^20^. Both SDF-1α and EGF are also well known to initiate chemotaxis of breast cancer cells in the transmembrane migration assay^21^. In these experiments, the induced a.c. electric field was produced by applying the same 20 Vpp, 100 kHz sawtooth shaped voltage waveform described earlier (Fig. 2a).

**Figure 4:**
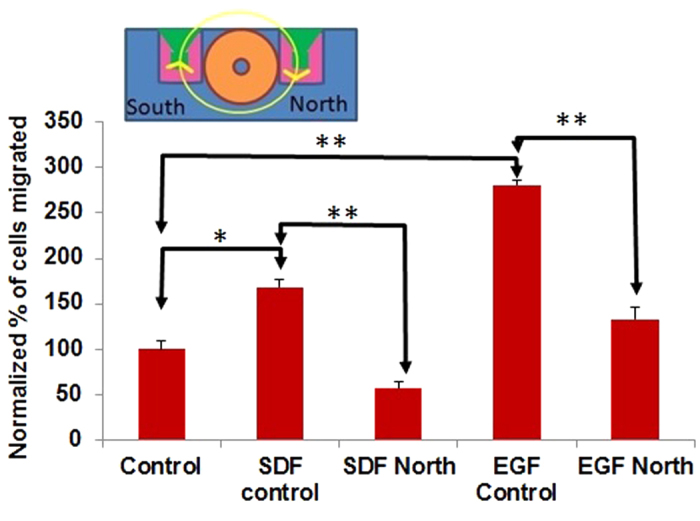
Summary of results of SCP2 cell migration in modified transmembrane assay showing the effects of induced E fields with and without chemokines/growth factors (SDF-1α or EGF) after 8 hours (N=3) Inset shows schematic end view of apparatus showing the direction of the induced field for ~60% of the 10 μs period. The function generator drives the coil with a 20 Vpp sawtooth shaped voltage transient at 100 kHz duty cycle. **Control:** Control without induced E fields or chemokines/growth factors in our modified transmembrane assay. **SDF control:** Control in our modified transmembrane assay with a Transwell insert, without induced E fields but in the presence of chemokine SDF-1α in the lower chamber (Control vs. SDF control: p =0.015). **SDF North:** Migration is hindered on the “North” side of the coil, where for the majority (60%) of the 10 μs period, the induced E field is in the direction of migration (i.e. directed downward), *even* in the presence of the chemokine SDF-1α (SDF control vs. SDF North: p =0.001). **EGF control:** Control in our modified transmembrane assay with a Transwell insert, without induced E fields but in the presence of growth factor EGF in the lower chamber (Control vs. EGF control: p =0.001). **EGF North:** Migration is hindered (relative to the case where there is no E field) on the “North” side of the coil, where for the majority (60%) of the 10 μs period, the induced E field is in the direction of migration (i.e. directed downward), in the presence of growth factor EGF (EGF control vs. EGF North: p =0.001).

We find that even in the presence of chemokines/growth factors such as SDF-1α and EGF, the induced electric fields on the “North” side hinder migration of SCP2 cells relative to migration levels without the field (SDF control versus SDF North, p=0.001; and EGF control versus EGF North, p=0.001, Fig. 4). Chemotaxis control experiments were also performed separately with chemokine SDF-1α (Control versus SDF control, Fig. 4, p=0.015) and growth factor EGF (Control versus EGF control, Fig. 4, p=0.001). Several key observations are immediately evident from these results (Fig. 4). First, chemotaxis is well enabled in the presence of chemokine SDF-1α and growth factor EGF in our modified transmembrane assay. Second, even in the presence of this chemokine and growth factor, the weak induced electric field applied in the direction of migration successfully *hinders* the migration of SCP2 cells compared to the corresponding cases of chemotaxis in the presence of SDF-1α or EGF alone. This is a significant result since recent works have shown that disruption of the SDF-1α signaling pathway can prevent metastasis and improve the ability of other treatment modalities (radiation or chemotherapy) to attack the tumor^18^,^20^,^22^.

### Modified apparatus enables visualization of actin filaments under induced electric fields

**Figure 5:**
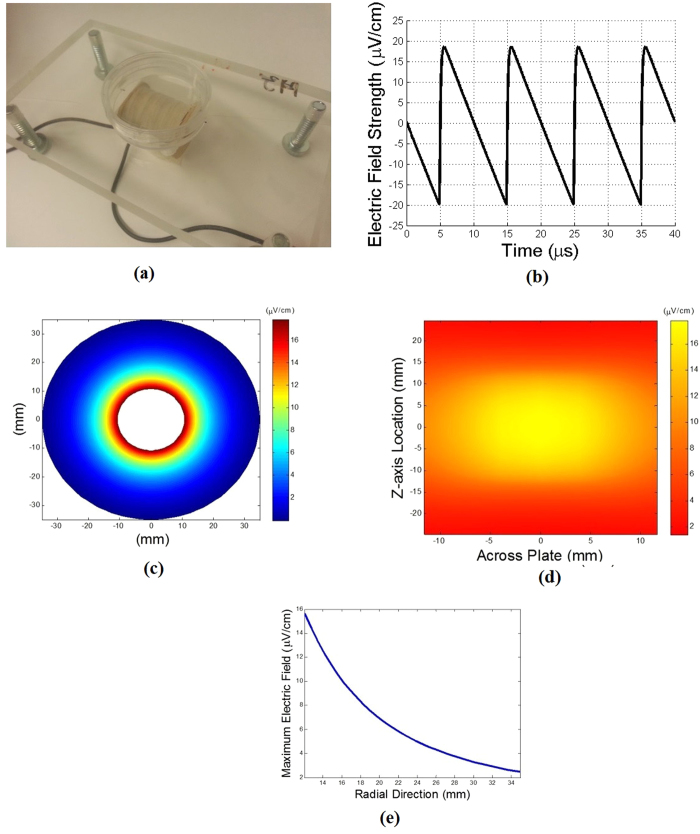
Apparatus for visualizing actin filaments under induced electric fields and results of experiments. The current through the coil is driven by a 20 Vpp sawtooth shaped voltage waveform at 100 kHz duty cycle. **(a)** Apparatus showing coil used in actin imaging experiments, in a 3-D printed holder and placed underneath a culture plate. **(b)** Induced electric field versus time showing shape of the field in 10 μs intervals. Note that this coil design yields maximum electric field strengths of ~20 μV/cm, and is approximately symmetric within the 10 μs period (i.e. on for equal duration leftward and rightward). **(c)** Contours of induced electric field when viewed from one end of the coil at the instant when the maximum induced E field is ~20 μV/cm. **(d)** Contour plot of the induced E field at the bottom of the culture plate, at the instant when its maximum value is ~20 μV/cm. Note that the induced E field is fairly uniform spatially over a region approximately 1 cm × 1 cm. Cells are typically plated in the center of the plate. **(e)** Variation of the induced E field versus (radial) distance away from the coil, at the instant where its maximum value is ~20 μV/cm. Since the thickness of the bottom of culture plates is typically ~1 mm, it is important to keep this variation in mind as one designs coils to exert a particular value of the induced E field at specific locations of a culture plate.

The actin cytoskeleton is known to play an important role in cell migration, especially in transmitting force through adhesion complexes to the substrate. Visualization of actin filaments can therefore help identify so called leader cells^23^ and expose any effects of induced electric fields on the internal cell machinery involved in migration. In aid of observing the actin cytoskeleton, a separate holder assembly (Fig. 5a, Supplementary Fig. S5) can be easily constructed to orient an electromagnetic coil in such a way as to place a single-well culture plate or a multi-well culture plate on top of a horizontal coil (Supplementary Fig. S6). In such a configuration, the induced electric field can be calculated for the 20 Vpp, 100 kHz sawtooth shaped voltage waveform discussed here (Fig. 5b–d). Depending on the coil diameter, shape and size, the field can be made uniform over a desired region of the culture plate. Our method is particularly well suited to being used in conjunction with the scratch assay^24^.

**Figure 6:**
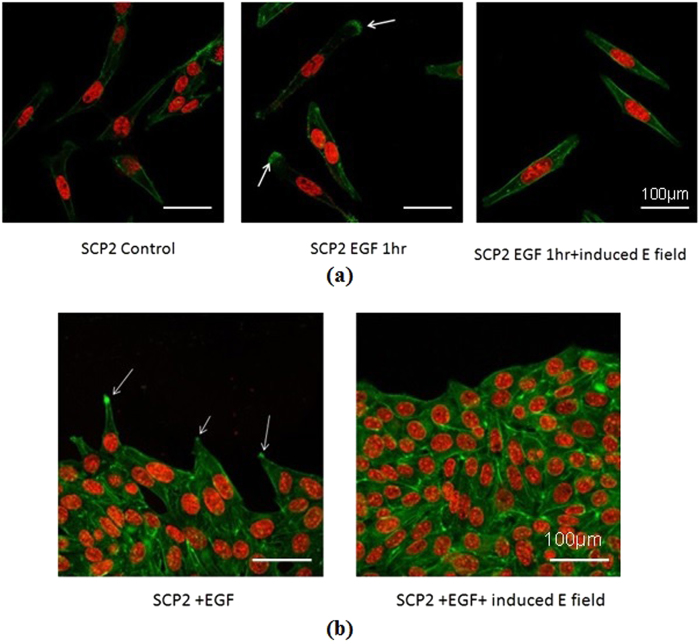
Visualization of actin filaments by fluorescence microscopy. **(a) (left panel)** actin cytoskeleton in SCP2 cells in the absence of both EGF and induced electric fields. **(a) (center panel)** actin cytoskeleton in SCP2 cells in the presence of the growth factor EGF. The white arrows indicate regions of polymerization of actin filaments signifying cellular movement or preparation for movement in response to the growth factor. **(a) (right panel)** actin cytoskeleton in SCP2 cells in the presence of EGF and an induced electric field. Note the polymerization of actin filaments within the cell filopodia in the center panel (indicated by white arrows) which are absent in the left and right panels. In the presence of the induced electric field, there is no preferential direction in the formation of the actin bundles. **(b) (left panel)** Edge of a contiguous layer of SCP2 cells in the presence of EGF, showing actin polymerization (indicated by white arrows) in some cells (so called “leader” cells) as they migrate or prepare to migrate. **(b) (right panel)** Edge of a contiguous layer of SCP2 cells in the presence of EGF and induced electric fields. Note there is no preferential direction for formation of actin bundles. Green and red represent F-actin and nucleus staining, respectively, where the F-actin is detected phalloidin-fluorphore (Alexa Fluor 568) conjugate.

We have visualized actin filaments that form the cytoskeleton of the SCP2 cells and play a crucial role in cell migration, when induced electric fields are applied in a non-contact manner as described earlier (Fig. 6). The images are quantified to determine the actin filament distribution within the cell as represented by the fluorescence intensity (Supplementary Methods). Details of the coil used in these experiments are given elsewhere (**Methods**). SCP2 cells are plated on a single-well, 60 mm diameter culture plate, allowed to migrate freely for an hour, and then fixed and stained with phalloidin-fluorophore conjugate for visualization of the actin filaments (Fig. 6a, **left panel**). SCP2 cells were incubated with EGF for one hour in the presence and absence of induced electric fields and the actin filaments stained with phalloidin-fluorophore conjugate were visualized by confocal microscopy (Fig. 6a**, middle and right panels**). These images have also been quantified after importing into MATLAB (Supplementary Figs. S12-S14). In the control case (no electric field and no chemokine), there is little visible polymerization of actin filaments and no discernible preferential direction of formation of filopodia (Fig. 6a **left panel**, Supplementary Fig. S12). In contrast, in the presence of the growth factor EGF, polymerization of actin filaments can be observed at one end of some cells (Fig. 6a **middle panel**, Supplementary Fig. S13). When an electric field is induced in the presence of EGF, actin polymerization can be seen throughout the cells with no preferential direction and which inhibits formation of filopodia (Fig.6a **right panel**, Supplementary Fig. S14). These effects are also apparent when a contiguous layer of SCP2 cells is formed (Fig. 6b). The so-called “leader” cells at the edge of the contiguous layer can be seen to respond to the growth factor EGF (Fig. 6b, **left**), while in the presence of both EGF and the induced electric field, no directional polymerization of actin is evident (Fig. 6b, **right panel**).

### Weak a.c. electric fields hinder chemotaxis of “normal” breast epithelial cells

Experiments on combined chemotaxis and electrotaxis were performed with MCF10A cells in the same modified transmembrane assay in order to examine the effects of the induced a.c. electric field in hindering chemotaxis of non-transformed cells. MCF-10A cells are a non-transformed epithelial cell line derived from human fibrocystic mammary tissue^17^. These cells are considered “normal” breast epithelial cells as they have a karyotype that is nearly diploid, are dependent on externally supplied growth factors for migration, and lack the ability to form tumors in nude mice. The growth factor EGF was used as an exogenous agent to induce MCF-10A cells to migrate in our modified transmembrane assay, as in the case of the SCP2 cells. No migration of MCF-10A cells was observed without EGF in the bottom chamber. As in the previous experiments with SCP2 cells, the induced a.c. electric field was produced by applying the same 20 Vpp, 100 kHz sawtooth shaped voltage waveform (Fig. 2a). Results with the induced electric field and in the presence of EGF were then compared to those of the control cases in the presence of EGF and no induced electric field.

The experimental results with the MCF-10A cells are summarized in Supplementary Figs. S15-S16. It can be seen that the MCF-10A cells do not migrate without the presence of the growth factor EGF. Furthermore, the induced electric fields on the “North” side hinder migration of MCF-10A cells relative to migration levels without the field in the presence of EGF (Coil North +E +EGF (4) versus −E +EGF (3), p=0.002; Supplementary Fig. S16). In contrast, induced electric fields on the “South” side do not affect MCF-10A cell migration in a statistically significant manner (data not shown).

## Discussion

A new method has been presented for inducing electric fields by electromagnetic induction (according to Faraday’s Law) and driving electrotaxis without the need for electrodes in contact with the media containing cell cultures. This method has been applied and demonstrated on our modified transmembrane assay commonly used for studying chemotaxis. The modification to the existing transmembrane assay consists of custom made glass wells (Fig. 1, Supplementary Fig. S1) that are designed to incorporate commercially available membrane inserts, placed on either side of an in-house designed and fabricated coil (Fig. 1a), and placed on a holder that is fabricated using 3-D printing technology (Supplementary Fig. S2). Our method can be applied to other cell migration assays such as the scratch assay^24^. By changing the length and diameter of the coil and associated holder, our method is also compatible with commercially available multi-well culture plates (Supplementary Fig. S6).

Experiments in our modified transmembrane assay using a single cell population SCP2 isolated from the MDA-MB-231 breast cancer cell line show that application of weak induced electric fields (on the order of ~1 μV/cm) is able to mitigate normal migration of these cells when the electric field is applied in the direction of migration. Moreover, SCP2 cell migration is also hindered in the presence of these weak a.c. induced electric fields, in the presence of the well-known chemokine SDF-1α and growth factor EGF. To our knowledge, this is the first time that such low-level electric fields (as low as *six orders of magnitude smaller* than previously reported) have been shown to have an effect on electrotaxis. No negative effects of the induced electric fields on the cells have been observed. In fact, experiments in which the induced electric field was applied for 8 hours to hinder SCP2 cell migration revealed that the cells continued to migrate normally when the electric field was shut off. We have reported effects of induced electric fields on inhibition of formation of filopodia and directional polymerization of actin, but the underlying mechanisms responsible for the molecular events are yet to be elucidated.

Experiments have also been performed on the non-transformed human mammary epithelial cells MCF-10A in our modified transmembrane assay. These cells do not normally migrate unless growth factors are externally supplied. Results from these experiments also show that application of weak induced electric fields is able to hinder their migration in the presence of growth factor EGF and when the field is applied in the direction of migration. These experiments show that the platform for applying induced electric fields presented here is applicable to different cell lines, both non-transformed and transformed.

The usefulness of the method presented in this paper may extend beyond using the modified transmembrane assay for quantifying the degree of metastasis of a particular cell line, or for studying electrotaxis when subjected to an a.c. field in a non-contact manner and in the presence of chemokines. The non-contact manner in which the E-field is applied may be useful in inhibiting metastasis, or in orchestrating wound healing *in vivo*. By varying the direction and spatial extent of the induced electric field, the approach presented here can enable different cells (e.g. keratinocytes, fibroblasts, endothelial cells, and macrophages) to migrate at different times during the wound healing process resulting in accelerated healing beyond just the superficial layers.^2^,^7^,^9^,^25^ Application of electric fields over periods of hours and days is also physiologically relevant in the treatment of cancers. So called tumor treating fields (TTF) have been successfully used to treat recurrent glioblastoma (GBM) and extend patient survival^26^. While the mechanism of action of TTFs may be different than the method presented here (the induced a.c. electric fields in this work are up to 6 orders of magnitude smaller), cell migration may be affected in both approaches. Finally, it is anticipated that by combining the ability to simultaneously study chemotaxis and electrotaxis using our modified transmembrane assay, new combinations of treatment strategies and drugs may be identified or ruled out earlier in the drug discovery screening process by revealing undesirable effects^13^.

## Methods

### Fabrication of holder for modified transmembrane assay experiments

The custom made glass wells fabricated using the methods described elsewhere (Supplementary Methods) are only identical in dimension up to fractions of a millimeter (Supplementary Fig. S1). Consequently, it is recommended that the holder be designed using computer-aided design (CAD) methods and fabricated after the glass wells have been made. Once the dimensions of the glass wells have been determined (Supplementary Fig. S1), a CAD drawing is developed using the commercially available software SolidWorks (Supplementary Fig. S2). The holder is then printed using plastic material according to the specifications on the CAD drawing on a 3D printer (Stratasys Fortus 400 MC). Since the outer radius of the coil may vary along its length due to unevenness of the windings, it is recommended that the middle channel where the coil is to reside be made of the smallest outer diameter of the coil so that specific locations in the channel may be ground manually to ensure that the coil fits in the center channel properly. The depth of each well in the holder is designed so as to ensure that the membrane is positioned exactly at the height corresponding to the centerline of the coil (Fig. 1b).

### Fabrication of coil for modified transmembrane assay experiments

The electromagnetic coil used to generate the induced electric fields across the transmembrane inserts consists of multiple windings (35 layers, ~159 turns per layer) of insulated 32 AWG (0.268 mm diameter with insulation or 0.202 mm diameter bare) wire wound around a glass rod. The inner diameter of the coil is 3 mm, the outer diameter is 1.4 cm, and its length is 10.5 cm. The coil resistance and inductance were measured using an LCR meter (Extech Instruments Model 380193) to be 50.45 Ω and 14.25 mH, respectively, at 1 kHz. The coil is placed at the center of the holder (Supplementary Fig. S2) with six custom made glass wells on either side (Fig. 1a,b). The wire gage, inner and outer diameters, number of turns, length, and number of layers in the coil can be varied depending on the type of experiment to be conducted. The duty cycle of the imposed voltage (which is 100 kHz for the results presented here) and its magnitude can also be easily changed. It is important to ensure that the function generator (Hewlett Packard 33120A 15 MHz in the present experiments) is able to drive sufficient current through the coil.

### Fabrication of custom glass wells and analysis of data

Fabrication of custom glass wells to accommodate transmembrane inserts, Analysis of induced electric fields in modified transmembrane assay experiments, Analysis of induced electric fields in visualization of actin filaments, and supplemental data on migration of MCF-10A cells with and without growth factor EGF and with and without induced electric fields, are described in the Supplementary Information section.

### Preparation of SCP2 cells

#### Cell migration

Low passage SCP2 cells were cultured in Dulbecco’s Modified Eagle’s Media (DMEM) containing 10% fetal bovine serum (FBS) and 5 U/mL penicillin, and 5 mg/mL streptomycin at 37 °C in a humidified culture incubator supplied with 5% CO_2_. To perform cell migration and actin filament imaging experiments, SCP2 cells were washed with serum free media three times and incubated with 0.1% FBS-DMEM media for 6 hours. The cells were detached from the culture plates by incubating in 1 mL of trypsin-EDTA for 2–4 min. The trypsin was neutralized by adding 2 mL of 0.1% FBS-DMEM. The cells were centrifuged at 1200 rpm for 5 min and re-suspended in 1 mL of 0.1% FBS-DMEM. The cells were counted using a hemocytometer. 1.5 × 10^5^ cells in 150 μL of media were placed in the top chamber of the modified transmembrane assay. The bottom chamber was filled with 600 μL of 0.1% FBS-DMEM with or without 100 ng/mL of chemokine (SDF-1α) or growth factor (EGF). After allowing 8 or 16 hours of incubation in the modified transmembrane assay, the cells that migrated to the other side of the Transwell membrane in the top chamber were stained with Hema 3 stain kit (Fisher Scientific, 122–911) according to the manufacturer’s instructions. The stained cells were then photographed with a Zeiss microscope attached to a camera. The migrated cells were counted in 5 representative fields. As an illustration, 4 representative fields for a control case and a case with the induced electric field are shown in Supplementary Fig. S3.

### Preparation of MCF-10A cells

#### Cell migration

MCF10A cells were cultured in Dulbecco’s Modified Eagle’s Media (DMEM) F12 containing 5% horse serum (HS), 20 ng/ml epidermal growth factor (EGF), 0.5 mg/ml hydrocortisone, 100 ng/ml cholrea toxin, 10 μg/ml insulin and 5 U/mL penicillin, and 5 mg/mL streptomycin at 37 °C in a humidified culture incubator supplied with 5% CO_2_. MCF10A cells were prepared for migration assay using 0.1% HS-DMEM-F12 media as described above. 1.5 × 10^5^ cells in 150 μL of media were placed in the top chamber of the modified transmembrane assay. The bottom chamber was filled with 600 μL of 0.1% HS-DMEM-F12 with or without 50 ng/mL EGF. After allowing migration for 16 hours, cells were stained, photographed and counted as described for the preparation of the SCP2 cells.

### Fluorescence microscopy

For the actin imaging experiments, the SCP2 cells were cultured in 60 mm culture dishes (Falcon, 353001) overnight in 10% FBS-DMEM and subsequently incubated in 0.1% FBS-DMEM for at least 6 hours. In another experiment to make a contiguous layer of cells, a straight wound (scratch) was created by the tip of 200 μL pipette tip. The wound was aligned on top of the coil axis to observe the effects of our induced electric field on EGF-induced actin polymerization. The cells were incubated in EGF (100 ng/mL) for 1 hour in the presence or absence of an induced electric field. Subsequently, the cells were washed with ice cold PBS and fixed with 4% paraformaldehyde. Further, the cells were permeabilized by 0.1% triton X-100 for 15 min and stained with Phalloidin conjugated with the fluorophore Alexa Fluor 568 (1:300X) (Molecular Probes) for 1 hour. Finally, the cells were mounted with VECTASHIELD hard set mounting media with DAPI (Vector labs) and visualized using an Olympus FV1000 confocal microscope.

### Statistical Analysis

To achieve statistical significance, in some cases three independent experiments consisting of 3 wells each were performed and representative data presented here. In other cases, two independent experiments consisting of two wells were performed. The data were computed as mean ± SD. Group means were compared by using the Student *t* test and p < 0.05 was considered as significant. Statistical analysis was performed with Microsoft excel (Microsoft Corporations, USA). Statistical significance is denoted in the figures by ‘*’ (0.01 ≤ p < 0.05), ‘**’ (0.001 ≤ p < 0.01), and ‘***’ (p < 0.001). Where sample sizes (N) are indicated, these denote results from independent experiments. For example, N=3 refers to three independent experiments measuring migration on for example the “North” and includes the 3 wells on that side of the coil.

## Additional Information

**How to cite this article**: Ahirwar, D. K. *et al.* Non-contact method for directing electrotaxis. *Sci. Rep.* **5**, 11005; doi: 10.1038/srep11005 (2015).

## Supplementary Material

Supplementary Information
