# Supplementary material for: Non-contact method for directing electrotaxis
Source: Sci Rep. 2015 Jun 9;5:11005. doi: 10.1038/srep11005 (PMC4460731; doi:10.1038/srep11005)
Supplement: Supplementary Information [file srep11005-s1.pdf]

Supplementary Information

Non-contact method for directing electrotaxis

Dinesh K. Ahirwar, Mohd W. Nasser, Travis H. Jones, Emily K. Sequin, Joseph D. West, Timothy L. Henthorne, Joshua Javor, Aniruddha M. Kaushik, Ramesh K. Ganju, and Vish V. Subramaniam

Contents:

Supplementary Methods
Supplementary Results
Supplementary Figures S1- S16
Supplementary References R1-R4

SUPPLEMENTARY METHODS

Fabrication of custom glass wells to accommodate transmembrane inserts

The glass wells (**Fig. 1b and Supplementary Fig. S1**) in which the transmembrane inserts can be placed were made from 14.6mm I.D. x 17mm O.D. borosilicate glass tubing (C.O.E. $32x10^{-7}$). Using a fixed carbon roller adjusted to an angle of 120 degrees to the glass tubing as it turned being chucked in a lathe, a hand torch heated the material until a suitable working state of the glass was reached. At which time, air was introduced through the center of the closed end tube, expanding the molten material until it made contact with the graphite roller. The roller shaped the material into the desired conical shape, leaving a tubular opening on each end to be closed later. Following formation of the well shape, the larger end was closed and shaped flat, this surface being perpendicular to the wall of the stock tubing. The finished end was then fixed into the tail chuck of the glass working lathe to allow the part to be temporarily separated from the stock material from which it had been formed. The stock material was closed flat and perpendicular to its wall, leaving only a small opening in the form of a tabulation shape, to allow for expanding air inside the well to escape as the part was sized. The lower portion was finished to measure approximately 9 mm in length from the closed bottom up to the point where its diameter began to expand into the shape of a funnel. Once separated from the stock tubing, and leaving only a small tabulation, the part was annealed in a furnace at 570^0 C for 20 minutes, and allowed to naturally cool to room temperature.

The closed top was finished with a 16mm diameter hole cut close to one side or off center from the center of the part. This was to allow for the transmembrane insert to be placed as close to the inside wall of the glass well (and hence the outer surface if the coil) as possible. For consistency, a wooden block was drilled to accept the glass well lower diameter feature to a depth large enough to allow clearance for the temporary tabulation. This wooden block was then positioned and clamped onto the stage of a drill press, in a position adjusted to where the outside diameter of the cutter was 2.5 mm off center from the outside wall of the 14.6 mm x 17mm O.D. lower part of the well. Positioned in this manner, the cutter (made of brass) formed a hole off center in the desired position allowing the transmembrane insert to rest against the wall of the lower portion of the glass well. The glass well held in the wooden jig had modeling clay applied to the outside of the rim to contain a 100 grit carborundum cutting powder slurry mixed with water. The action of the brass mandrel turning against the flat glass surface of the top of the well with the cutting compound slurry proceeded to grind through over approximately 15 to 20 minutes. A pecking action was employed to reintroduce new cutting compound into the "groove" being formed.

The bottom was permanently closed after cleaning the part to remove the cutting compound and modeling clay. The part was chucked into the glass working lathe from the wider, flared end. As the part turned, it was warmed before a small torch was applied to heat the glass to a suitable working temperature allowing for the temporary tabulation to be removed. The part was then allowed to cool, before being turned in the opposite direction and chucked again into the lathe to apply a final finish on the 16mm diameter opening on the wider end. Finishing the open, 16 mm diameter end involved bringing the part back to a warmed condition before polishing the ground surface left from grinding the opening with the brass mandrel. Once warmed, a small hand torch was used to fuse the surface of the cut a little at a time until the entire circumference of the opening was "fire" polished. Care was taken not to overheat the

surfaces so as to not distort any other part of the well. The part was then flame annealed to a point safe from cracking before finally being oven annealed, as described earlier.

Analysis of induced electric fields in modified transmembrane assay experiments

A time-dependent magnetic induction ($\partial\vec{B}/\partial t$) must be present in order to induce an electric field in a non-contact manner. Such an induced electric field can be produced either by a constant magnitude magnetic field that is changing its direction versus time or by a magnetic field in a specific direction that is changing its magnitude with respect to time, or both. In this work, we have elected for the simplest approach which involves the latter.

The 20 Vpp, 100 kHz sawtooth shaped voltage waveform (**Fig. 2a**) imposed on the previously described electromagnetic coil (**Fig. 1a**) results in a time-dependent current flow through it. The current through the coil varies in time both in magnitude and direction, and is a complicated function of the inductance and intrinsic capacitance of the coil as well as its coupling with the function generator. Consequently, the resulting magnetic induction in cylindrical coordinates, $\vec{B}(r,z,t)$, and associated vector potential $\vec{A}(r,z,t)$ vary with time both in magnitude and direction, where r and z are the radial and axial coordinates measured from an origin located at one end of the coil along its centerline. The induced electric field $\vec{E}(r,z,t)$ can then be calculated from the vector potential, $\vec{A}(r,z,t)$:

$$\vec{E} = -\frac{\partial\vec{A}}{\partial t} \tag{1}$$

The time-dependent current through the coil can be measured using a sense resistance (a smaller resistance) connected in series with the coil in the circuit, and by measuring the time-dependent voltage drop across the 1.25 Ω sense resistance (**Supplementary Fig. S7**). Extreme care must be taken so as to ensure that no stray capacitances arising for example from BNC connectors corrupt the measurement. One way to verify the measurement, is to connect the sense resistance upstream of the coil and ensure that the same current profile versus time is obtained as when the sense resistance is connected downstream of the coil. It is also important to point out that use of a sense resistance for current measurement is only reliable at low values of the duty cycle (on the order of tens of kHz or lower) because of the unknown intrinsic coil capacitance which is difficult to quantify at high values of the duty cycle (e.g. at 100 kHz) due to leakage.

We adopt the following methodology for calculating the induced electric fields relevant to our experiments. The current through the electromagnetic coil is measured for an imposed sawtooth voltage waveform of 20 Vpp at 1 kHz using a 1.25 Ω sense resistance (**Supplementary Fig. S7**). A circuit element model (**Supplementary Fig. S8**) is then used to predict the current through the coil at 1 kHz, and validated against the current measurement using the sense resistance at 1 kHz. The model is used to infer the intrinsic coil capacitance (**Supplementary Fig. S8**), and then used to predict the coil conduction current for the experimental conditions where the imposed 20 Vpp sawtooth shaped voltage waveform is applied at 100 kHz. Once the time-varying current is calculated, the vector potential $\vec{A}(r,z,t)$ is calculated using an analytical solution for the vector potential at a point given a circular current winding of a given diameter at a specific location[R1]:

$$A_{\phi j} = \frac{\mu_0 I}{\pi}\sqrt{\frac{a_j}{m_j r}}\left[\left(1-\frac{m_j}{2}\right)K(m_j)-E(m_j)\right] \tag{2}$$

where $m_j = \frac{4a_j r}{\left[(a_j+r)^2+(z-l_j)^2\right]}$, K($m_j$) is the complete elliptic integral of the first kind[R2], E(m_j) is the complete elliptic integral of the second kind[R2], I is the current through winding j, a_j is the radius of the jth winding, r is the radial coordinate, z is the axial coordinate (with the origin taken along the centerline of the coil and at one end of the coil), and $A_{\phi j}$ is the contribution to the vector potential at (r,z) at time t due to current I(t) flowing in the jth winding. By taking the coil to be comprised of a perfectly stacked set of wire loops (windings) with different diameters and carrying the same current[R3], the vector potential at any point in space can be obtained as the superposition of the individual contributions from each loop of wire in the coil:

$$A_\phi = \sum_{j=1}^{N}\frac{\mu_0 I}{\pi}\sqrt{\frac{a_j}{m_j r}}\left[\left(1-\frac{m_j}{2}\right)K(m_j)-E(m_j)\right] \tag{3}$$

where N is the total number of windings in the coil (35 layers x 159 windings per layer = 5565). Note that in Eq.(3) the only time dependent quantity is the current I. The radial and axial components of magnetic induction are then calculated from:

$$B_r = -\frac{\partial A_\phi}{\partial z} = \sum_{j=1}^{N}\frac{\mu_0 I}{2\pi}\frac{(z-l_j)}{r\sqrt{(a_j+r)^2+(z-l_j)^2}}\left[-K(m_j)+\left(\frac{a_j^2+r^2+(z-l_j)^2}{(a_j-r)^2+(z-l_j)^2}\right)E(m_j)\right] \tag{4}$$

$$B_z = \frac{A_\phi}{r}+\frac{\partial A_\phi}{\partial r} = \sum_{j=1}^{N}\frac{\mu_0 I}{2\pi}\frac{1}{r\sqrt{(a_j+r)^2+(z-l_j)^2}}\left[K(m_j)+\left(\frac{a_j^2-r^2-(z-l_j)^2}{(a_j-r)^2+(z-l_j)^2}\right)E(m_j)\right] \tag{5}$$

and the induced electric field is given by:

$$E_\phi = -\frac{\partial A_\phi}{\partial t} = -\frac{\mu_0}{\pi}\left(\frac{dI}{dt}\right)\sum_{j=1}^{N}\sqrt{\frac{a_j}{m_j r}}\left[\left(1-\frac{m_j}{2}\right)K(m_j)-E(m_j)\right] \tag{6}$$

where $m_j = \frac{4a_j r}{\left[(a_j+r)^2+(z-l_j)^2\right]}$. Note that the induced electric field E_ϕ calculated from (6) varies with the radial coordinate r and axial coordinate z. For the case of rapidly changing transients, it is recommended that the derivative dI/dt in equation (6) be calculated using higher order accurate finite difference formulae such as a fourth-order accurate finite difference formula[R4].

The current through the coil is measured using a sense resistance of 1.25 Ω with the function generator supplying a 20 Vpp sawtooth waveform at 1 kHz (**Supplementary Fig. S9**). Also shown in the figure is the coil conduction current calculated using the circuit element model (**Supplementary Fig. S8**). The intrinsic coil capacitance was determined by parametric variation to be 30 nF using the measured values (at 1 kHz) of 50.45 Ω for the d.c. resistance and 14.25 mH for the inductance. Varying the intrinsic coil capacitance alters the magnitude of the spike whereas the inductance and resistance together shift the bowl shaped profile for all other times in the period (**Supplementary Fig. S9**). As can be seen, the agreement between prediction and measurement is excellent.

The circuit element model is used to predict the current at 100 kHz using the same values of inductance, resistance, and capacitance at 1 kHz. The resulting current as a function of time exhibits asymmetry over a period (**Supplementary Fig. S10**). The induced electric field is proportional to dI/dt (equation (6)) and is therefore also asymmetric over any given period (**Fig. 2b**).

Analysis of induced electric fields in visualization of actin filaments

For the purpose of visualizing actin filaments using phalloidin and fluorescence microscopy, the orientation of the coil has to be changed compared to the configuration used in the transmembrane assay experiments. The electromagnetic coil used to generate the induced electric fields in these experiments also consists of multiple windings (18 layers, ~ 67 turns per layer) of insulated 32 AWG (0.268 mm diameter with insulation or 0.202 mm diameter bare) wire. The inner diameter of the coil is 14.2 mm, the outer diameter is 2.332 cm, and its length is 2.47 cm. Measurements of the coil resistance and inductance using an LCR meter (Extech Instruments Model 380193) yielded 24.58 Ω and 12.17 mH, respectively, at 1 kHz. The coil is placed at the center of the holder with a standard 60 mm diameter culture plate on top (**Supplementary Fig. S11, Fig. 5a**). It must be pointed out that the physical characteristics of the coil (wire gage, diameter, number of turns, length, number of layers) given here are for the experimental results presented in this paper. These parameters as well as the duty cycle of the imposed voltage (which is 100 kHz for the results presented here) can all be easily changed depending on what effects one wishes to explore. It is important to ensure that the function generator (Hewlett Packard 33120A used in the present experiments) is able to drive sufficient current through the coil. In a similar manner, the holder can also be easily modified to accommodate a multi-well plate (**Supplementary Fig. S6**). Currents through the coil and the resulting magnetic inductions and induced electric fields can be calculated as described by equations (4) – (6) in the previous section for a 20 Vpp sawtooth voltage waveform at a duty cycle of 100 kHz (**Supplementary Fig. S11, Figs. 5b-5d**).

Analysis of actin filament distribution

Images obtained from phalloidin and fluorescence microscopy are imported into MATLAB (2014a, Mathworks, Inc., Massachusetts, U.S.A.). Individual cells are isolated and re-oriented so that their longest dimension is in the horizontal direction. Intensities are then separated into red, green, and blue so that the background and nuclei intensities may be filtered out and only the green fluorescence from the actin filaments is extracted. Actin fluorescence intensities are analyzed versus cell length (longer dimension being the length), and averaged. These post-processed images and average intensities for the isolated cells shown in **Fig. 6**, are shown in **Supplementary Figs. S12-S14**.

SUPPLEMENTARY RESULTS

Experimental results for "normal" breast epithelial cells (MCF-10A)

Experiments on combined chemotaxis and electrotaxis performed with MCF10A cells in the modified transmembrane assay utilized the growth factor EGF in the lower compartment of the modified transmembrane assay. After allowing 16 hours of incubation in the modified transmembrane assay, the cells that migrated to the other side of the Transwell membrane in the top chamber were stained with Hema 3 stain kit (Fisher Scientific, 122-911) according to the manufacturer's instructions. The stained cells were then photographed with a Zeiss microscope attached to a camera. The migrated cells were counted in 5 representative fields. As an illustration, representative fields are shown in **Supplementary Fig. S15**. Data for migration of MCF-10A cells with and without growth factor EGF and with and without induced electric fields, is shown in **Supplementary Fig. S16**. As can be seen from **Supplementary Figs. S15-S16**, MCF-10A cells hardly migrate without the presence of EGF (control in **Supplementary Fig. S16**), and the presence of an electric field in the direction of migration ("North" side) has no statistically significant effect in this case in the absence of EGF (Coil North +E -EGF in **Supplementary Fig. S16**). On the other hand, it can be seen that EGF strongly promotes migration of MCF-10A cells (-E +EGF in **Supplementary Fig. S16**) and our induced electric field hinders their migration (Coil North +E +EGF in **Supplementary Fig. S16**) with statistical significance (p=0.002) for –E +EGF versus Coil North +E +EGF in **Supplementary Fig. S16**).

Supplementary Figure S1

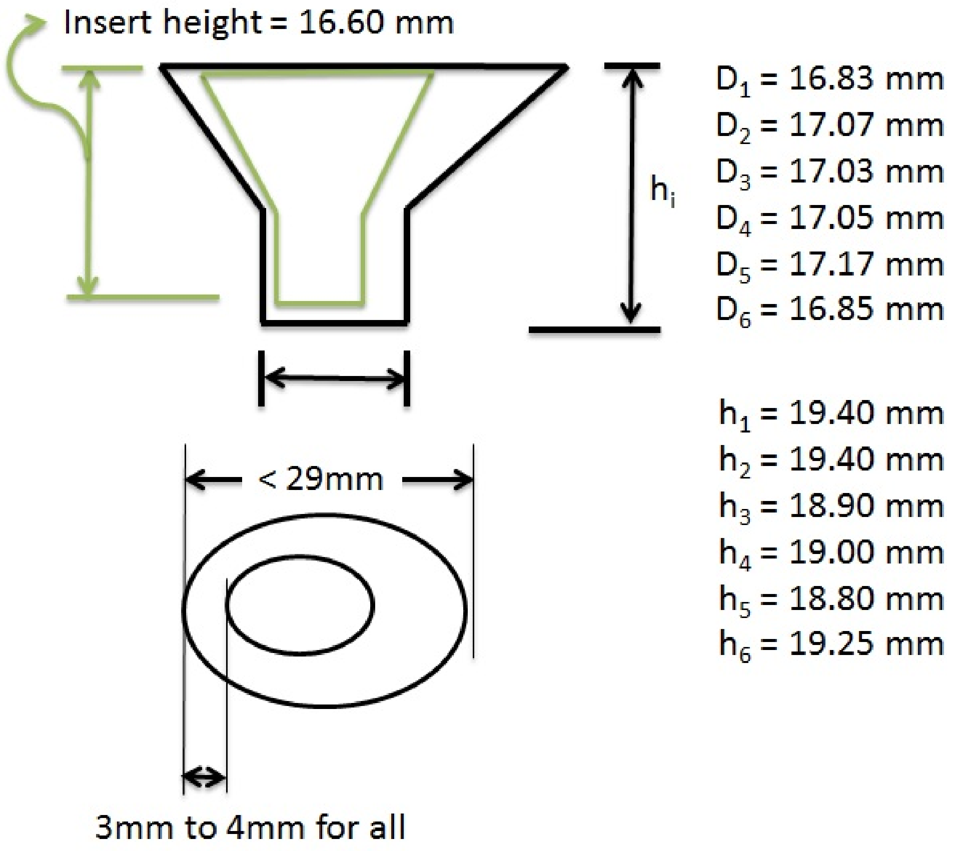

The glass wells are custom made to accommodate commercially available Transwell transmembrane inserts. The outer compartment and holder (**Supplementary Fig. S2**) are designed so that the membrane (the bottom surface of the inner funnel) is as close as possible to the outer surface of the coil, to within 1 mm.

Supplementary Figure S2

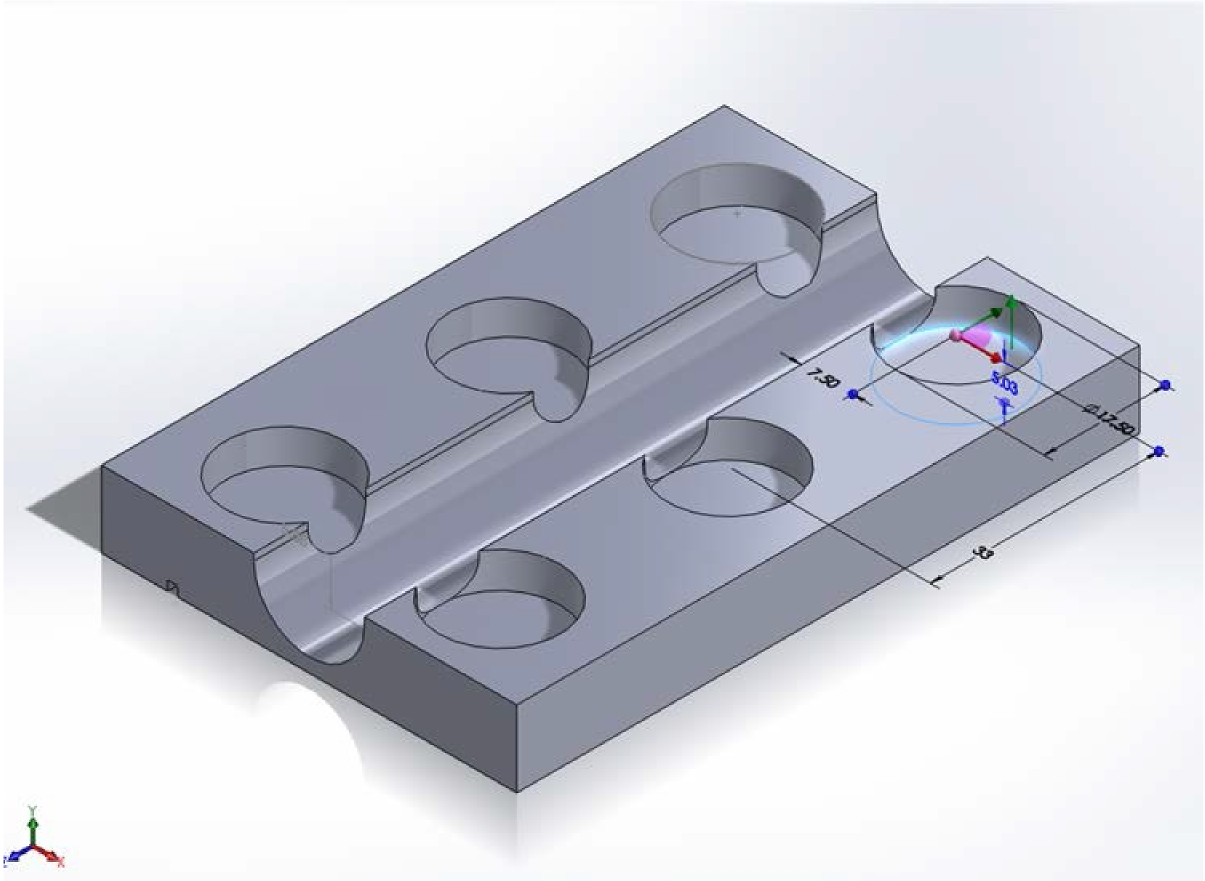

The holder is fabricated using 3-D printing technology after first developing a computer-aided design (CAD) drawing using the software SolidWorks. The circular wells in the holder have unique dimensions based on the exact dimensions of each glass well (**Supplementary Fig. S1**). The center channel allows the coil to be placed in the center so that there are 3 wells each on either side of the coil.

Supplementary Figure S3

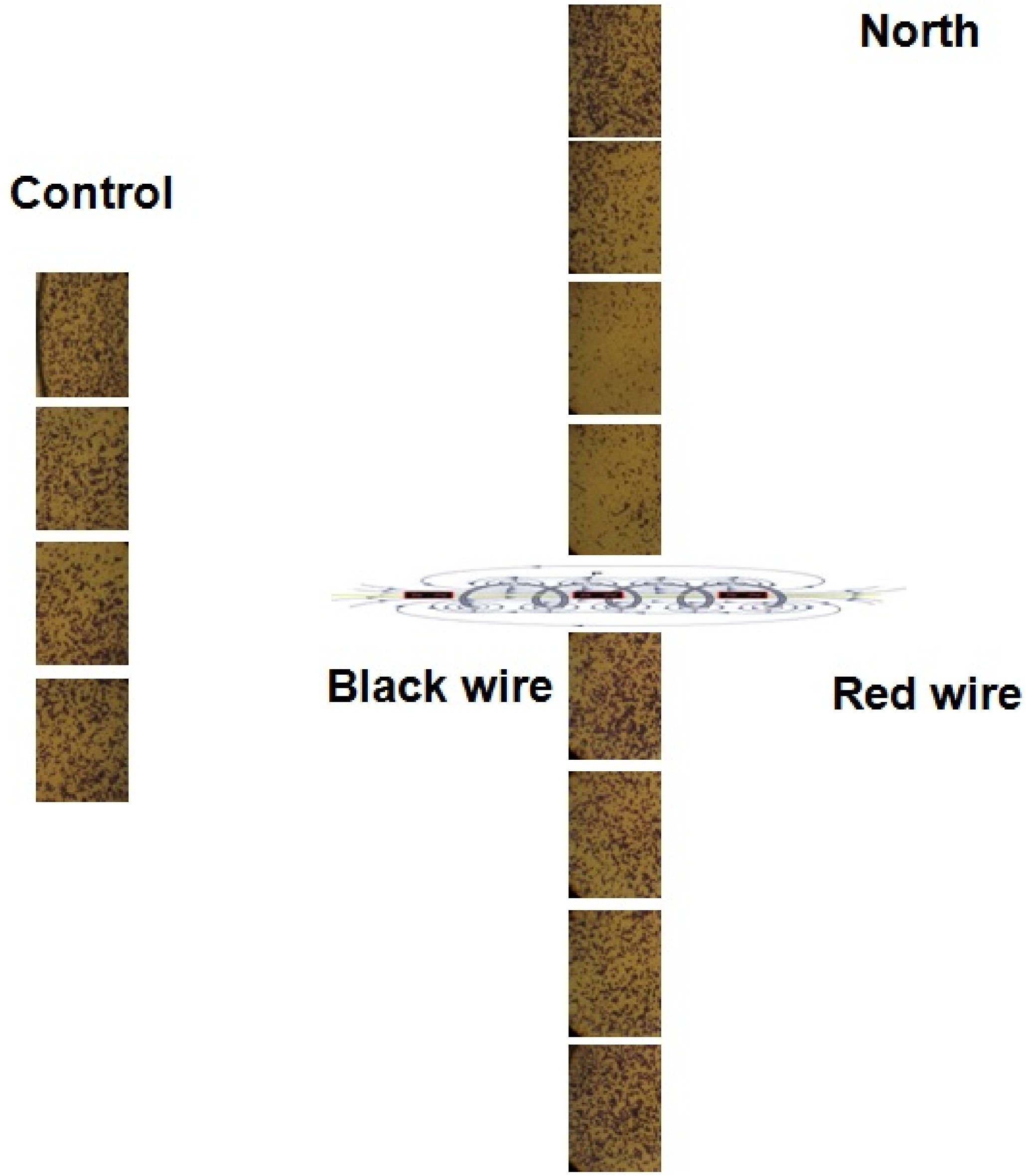

Representative fields (4 each) from the Transwell transmembrane assay and our modified transmembrane assay with SCP2 cells fixed and stained, showing how the cells are counted. The cells were allowed to migrate for 8 hours, and were fixed and stained using Hema-3 stain kit according to the manufacturer's instructions. The number of migratory cells per membrane was then measured using light microscopy by counting the total number of cells in each of 5 contiguous images taken at 20X magnification, spanning radially outward (5 fields) from the coil. The counts were used to determine the percentage of migration. Only a representative set of control images are shown here as there was no statistically significant difference between the control on the "North" and "South" sides.

Supplementary Figure S4

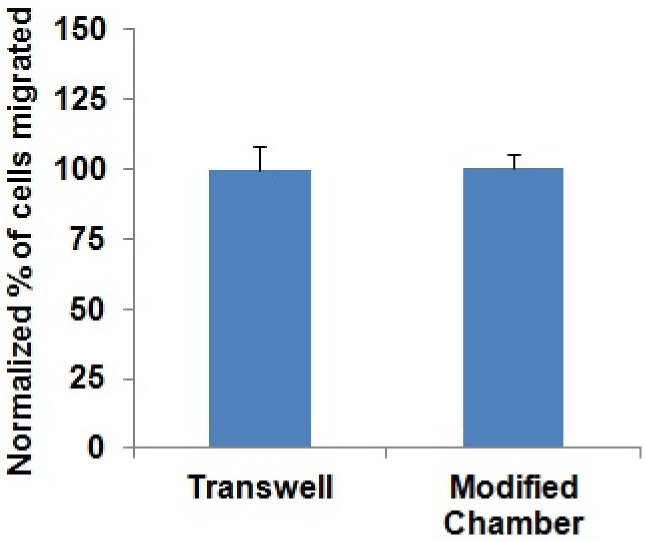

Our modified transmembrane assay (Fig. 1a and 1b) with the Transwell membrane inserts reproduces cell migration observed in the presently used multi-well plates with the same inserts. These cell migration experiments were performed over a period of 8 hours on SCP2 cells (N=3). No induced electric field was applied and the same media (0.1% FBS-DMEM) was used (p=0.993).

Supplementary Figure S5

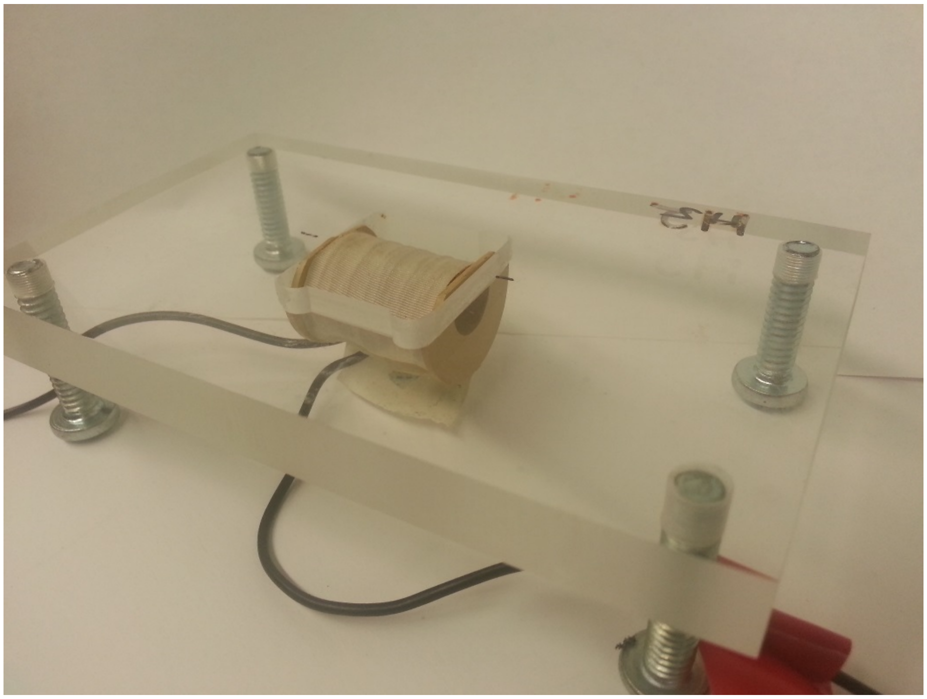

Schematic of modified holder, coil, and culture plate used in the experiments to image actin filaments using phalloidin and fluorescence microscopy. The holder is fabricated using 3-D printing technology after first developing a computer-aided design (CAD) drawing using the software SolidWorks.

Supplementary Figure S6

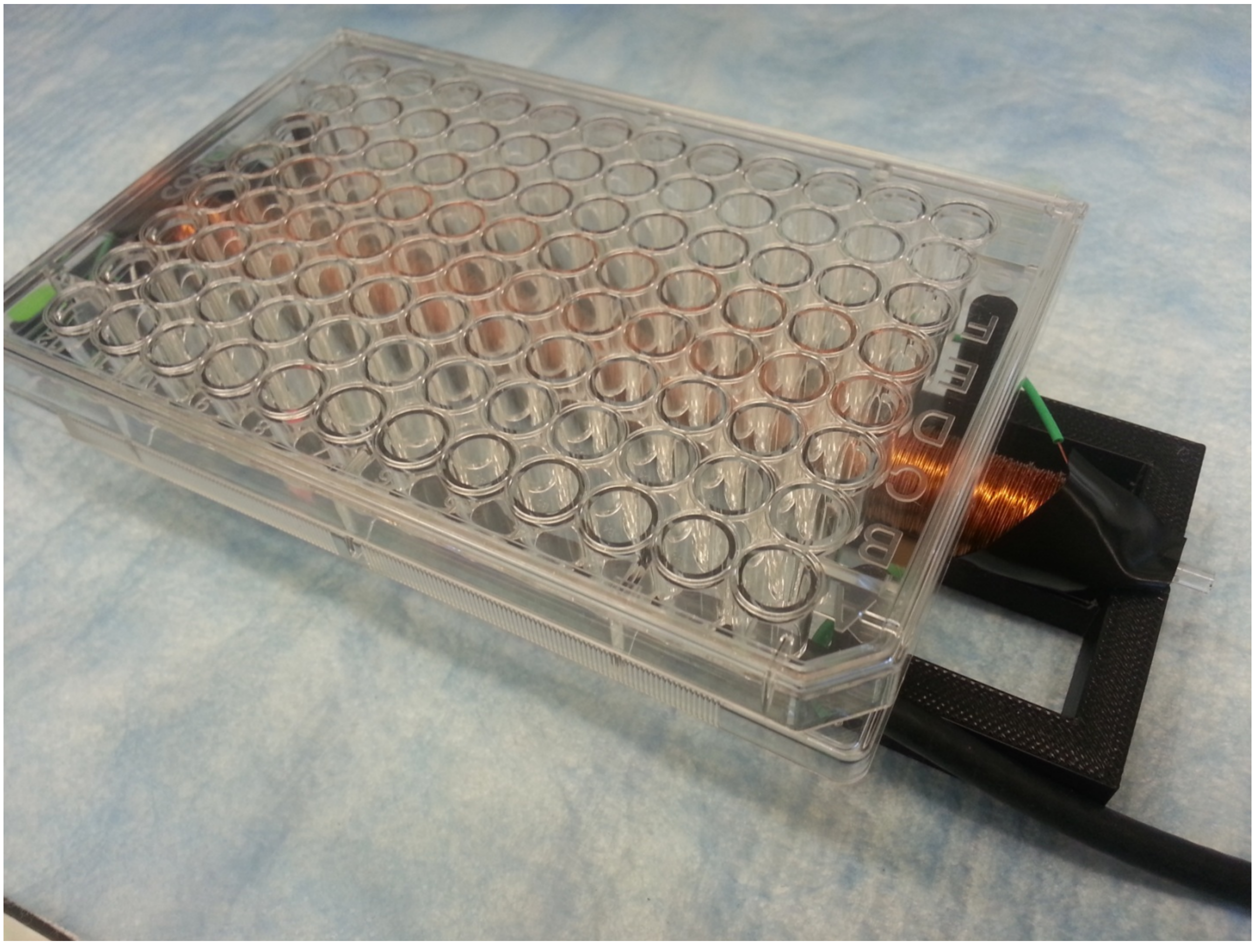

Photograph showing how the modified assembly for accommodating a culture plate (Supplementary Fig. S7) may be altered for a 96 well multi-well plate. The holder is fabricated using 3-D printing technology after first developing a computer-aided design (CAD) drawing using the software SolidWorks.

Supplementary Figure S7

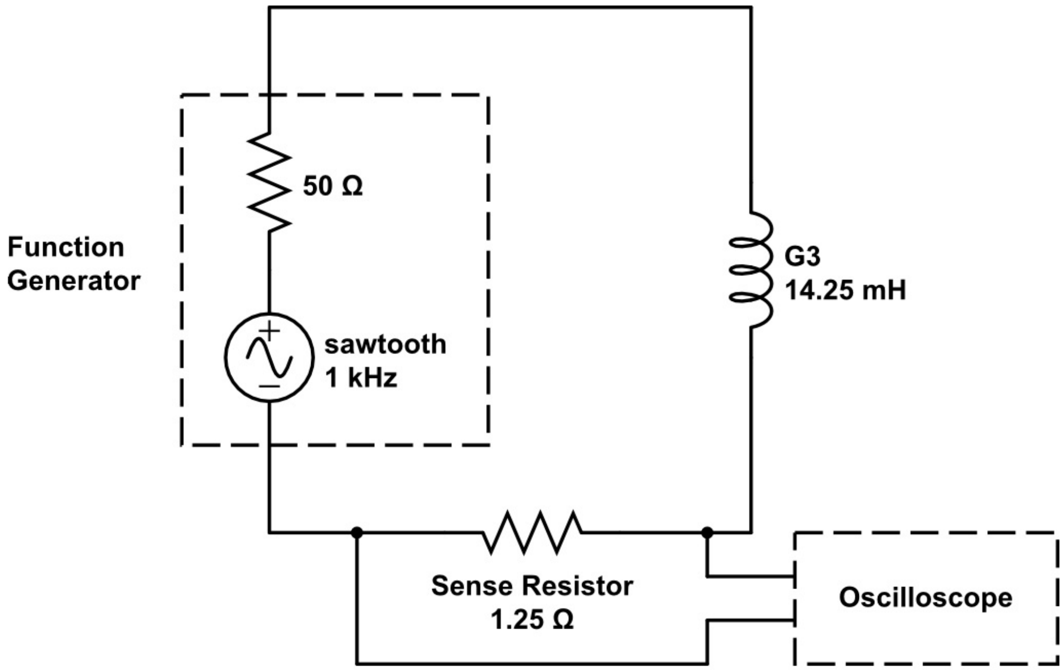

Circuit diagram showing the use of a sense resistor to measure the current through the coil. The coil has a d.c. resistance, inductance, and intrinsic capacitance (**Supplementary Fig. S8**). Consequently, measurement of the current in the circuit by placing the sense resistance downstream of the coil, and measuring the voltage drop yields the total current flow (sum of conduction and displacement currents through the coil). This measurement at 1 kHz allows the intrinsic capacitance to be inferred by comparing the measured voltage trace across the sense resistance with a voltage trace predicted by a circuit element model that simulates the coil as a resistor in series with an inductor, both of which are in parallel with a capacitor (**Supplementary Fig. S8**). This intrinsic coil capacitance is then used to calculate the conduction current at 100 kHz, relevant for the electrotaxis experiments reported here. It is important to mention that the current measurement described here is sensitive to stray capacitances that can arise from coax connectors (such as tees) placed at the oscilloscope. Therefore extreme care must be exercised to eliminate such sources of stray capacitance. Ultimately, a check of the measurement is the fact the current measured with the sense capacitance placed upstream of the coil should yield a similar current as when the sense capacitance is placed downstream of the coil.

Supplementary Figure S8

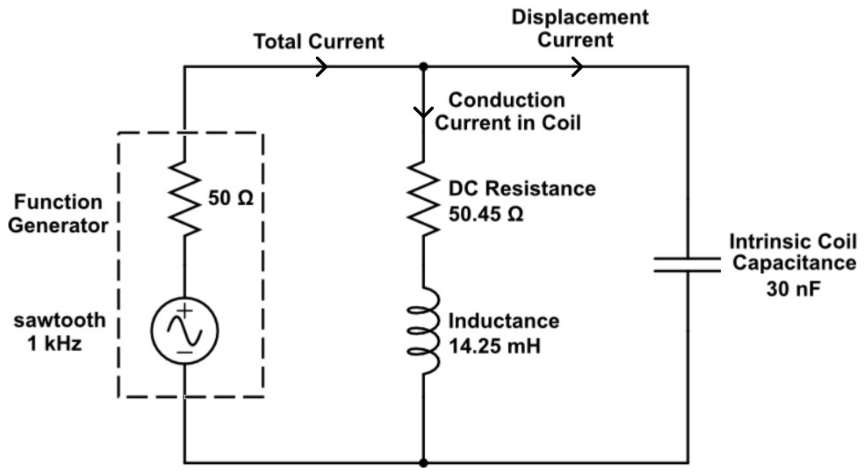

Circuit diagram showing the model used to predict the current through the coil. This circuit element model is used to predict the total current through the coil at 1 kHz, and compare with the current measurement using the sense resistance (**Supplementary Fig. S7**) at 1 kHz. This enables the intrinsic coil capacitance to be inferred (30 nF) by matching prediction with measurement at 1 kHz. This value of the intrinsic coil capacitance is then used to predict the conduction current through the coil at the experimental duty cycle of 100 kHz.

Supplementary Figure S9

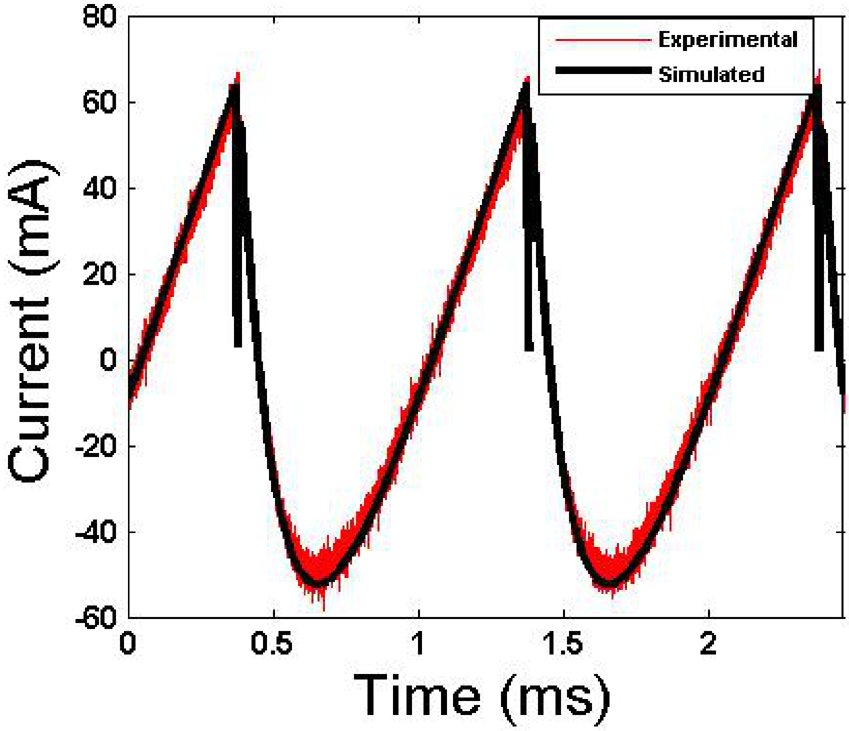

Total current through the electromagnetic coil used in the transmembrane assay experiments (measured in red, calculated in black) at a duty cycle of 1 kHz for a 20 Vpp sawtooth voltage waveform. By systematically varying only the intrinsic coil capacitance until the prediction matches the measurement yields a value of 30 nF. Note that the spike in the figure is due to the displacement current passing through the coil capacitance. This inferred value of the intrinsic coil capacitance is then used to predict the conduction current through the coil at 100 kHz.

Supplementary Figure S10

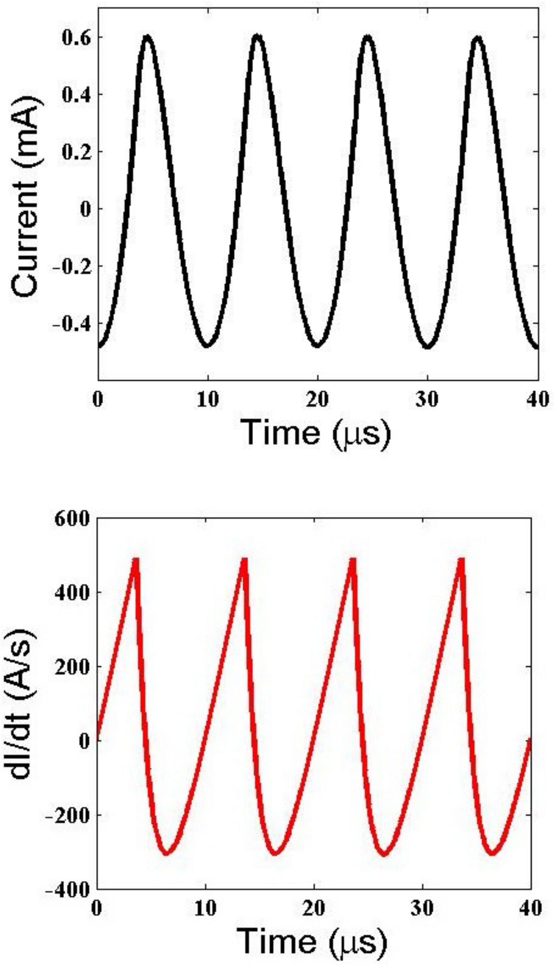

Predicted current (top) through the electromagnetic coil used in the transmembrane assay experiments, at a duty cycle of 100 kHz for a 20 Vpp sawtooth voltage waveform, and its derivative (bottom). It can be seen that the derivative of the current through the coil is asymmetric over a single period of the duty cycle so that the electric field is in the downward direction on the "South" and "North" sides of the coil for different durations (~40% on the "South" side and ~60% on the "North" side) (**Fig. 2b-2e**).

Supplementary Figure S11

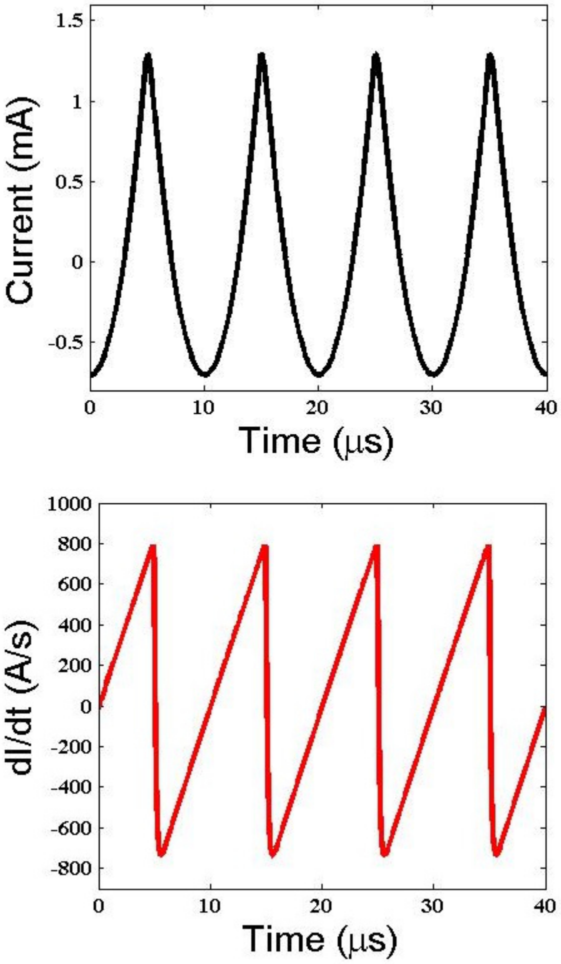

Predicted current through the electromagnetic coil used in the actin filament imaging experiments, at a duty cycle of 100 kHz for a 20 Vpp sawtooth voltage waveform. It can be seen that the derivative of the current through the coil is approximately symmetric over a single period of the duty cycle so that the electric field is in the leftward and rightward directions for different durations on the culture plate (when viewed from the top) (**Fig. 5b-5e**).

Supplementary Figure S12

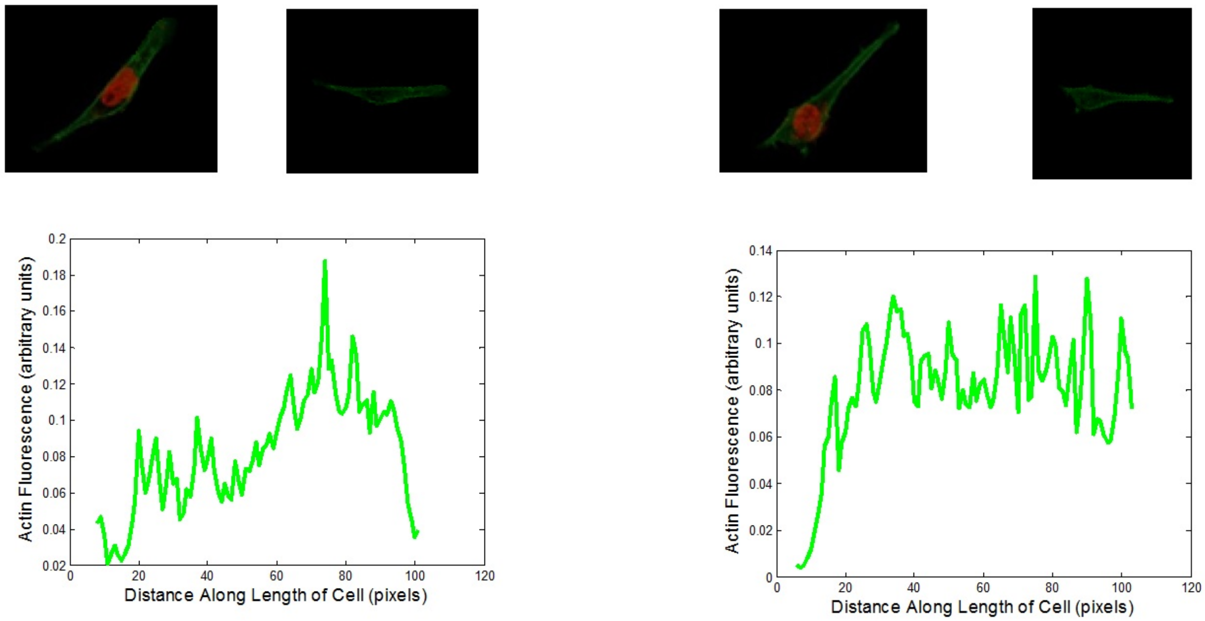

(A) **(B)**

Average intensity of actin fluorescence versus length along isolated cells shown in the left panel of Fig. 6a. The actin cytoskeleton in SCP2 cells in the absence of both EGF and induced electric fields is quantified here and averaged to show the distribution of fluorescence intensity versus length along the cell. Also shown in the upper right images of both panels (A) and (B) are the filtered images displaying only the actin cytoskeleton.

Supplementary Figure S13

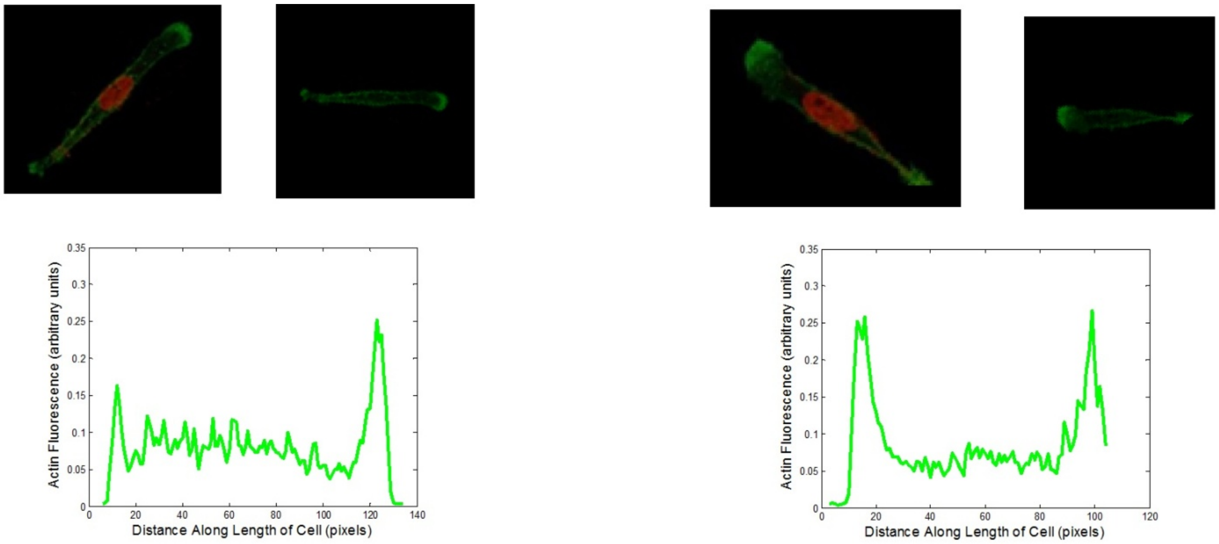

(A) **(B)**

Average intensity of actin fluorescence versus length along isolated cells shown in the middle panel of Fig. 6a. The actin cytoskeleton in SCP2 cells in the presence of EGF and without induced electric fields is quantified here and averaged to show the distribution of fluorescence intensity versus length along the cell. Also shown in the upper right images of both panels (A) and (B) are the filtered images displaying only the actin cytoskeleton.

Supplementary Figure S14

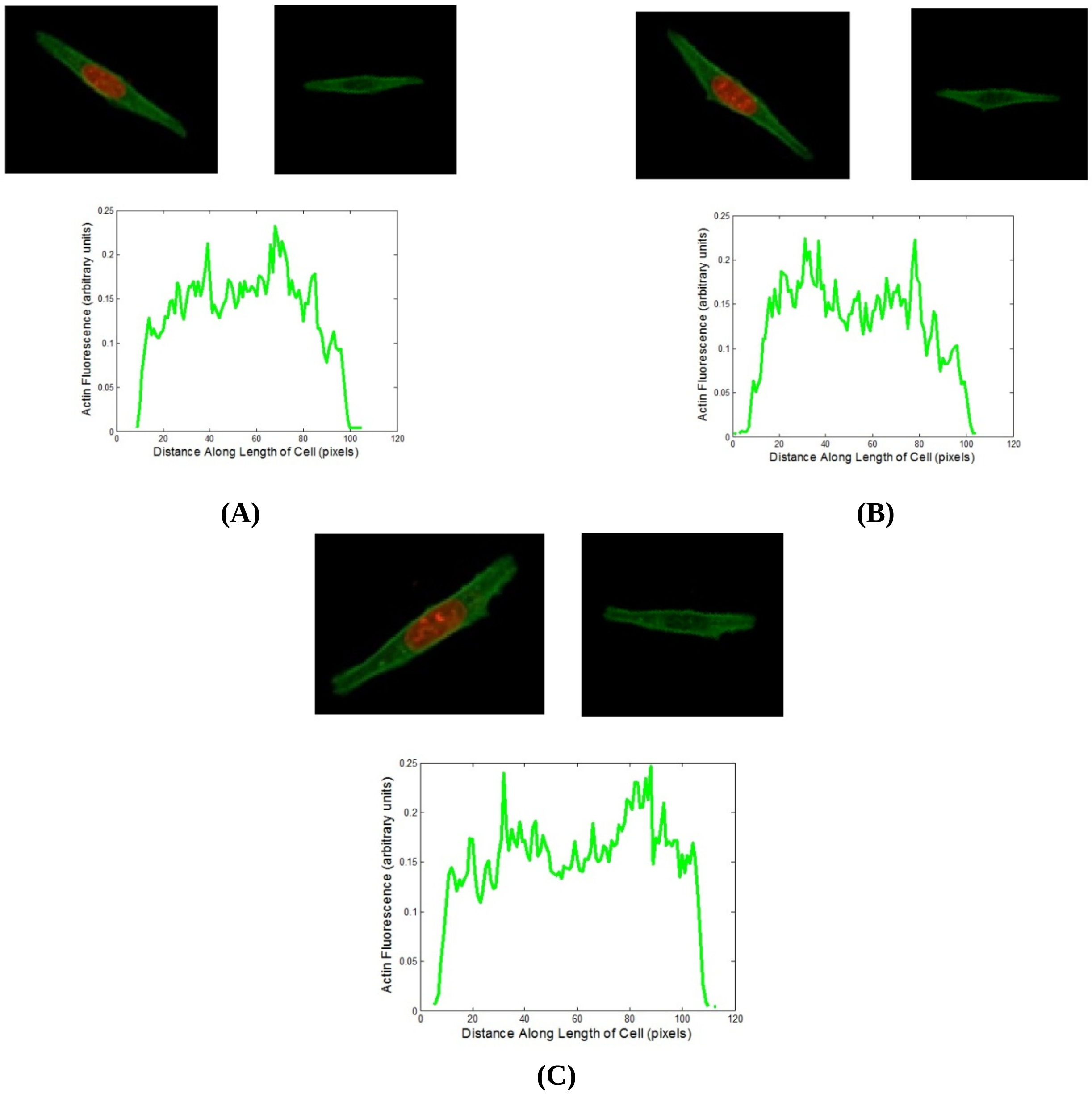

Average intensity of actin fluorescence versus length along isolated cells shown in the right panel of Fig. 6a. The actin cytoskeleton in SCP2 cells in the presence of both EGF and induced electric fields is quantified here and averaged to show the distribution of fluorescence intensity versus length along the cell. Also shown in the upper right images of panels (A), (B), and (C) are the filtered images displaying only the actin cytoskeleton.

Supplementary Figure S15

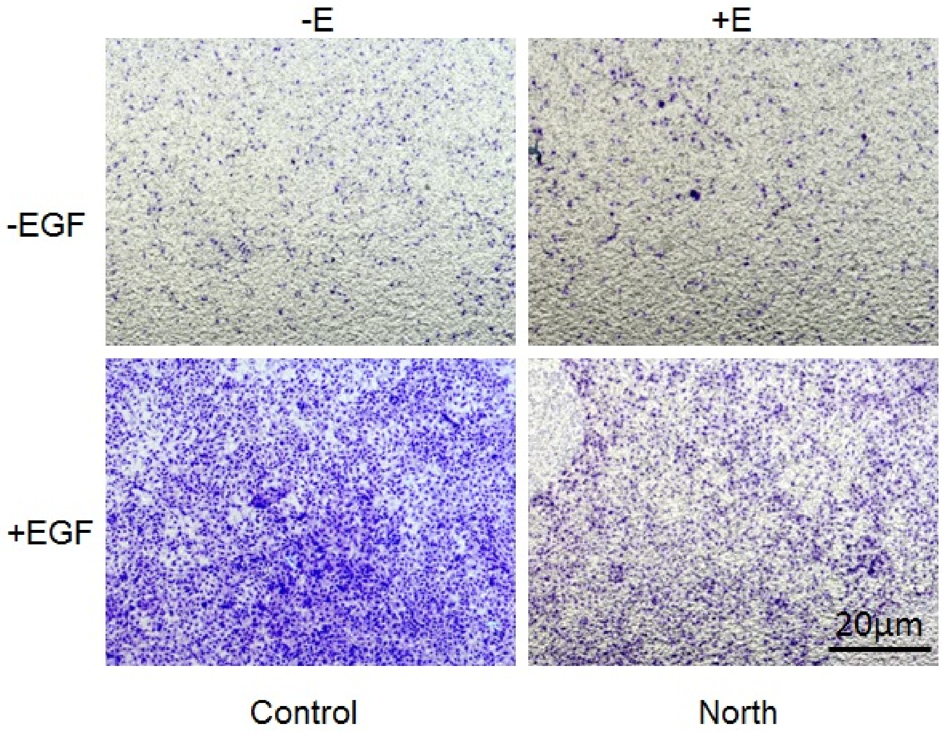

Representative fields from our modified transmembrane assay with a Transwell membrane with MCF-10A cells fixed and stained, showing how the cells are counted. The cells were allowed to migrate for 16 hours, and were fixed and stained using Hema-3 stain kit according to the manufacturer's instructions. The number of migratory cells per membrane was then measured using light microscopy by counting the total number of cells in each of 5 contiguous images spanning radially outward (5 fields) from the coil. The counts were used to determine the percentage of migration.

Supplementary Figure S16

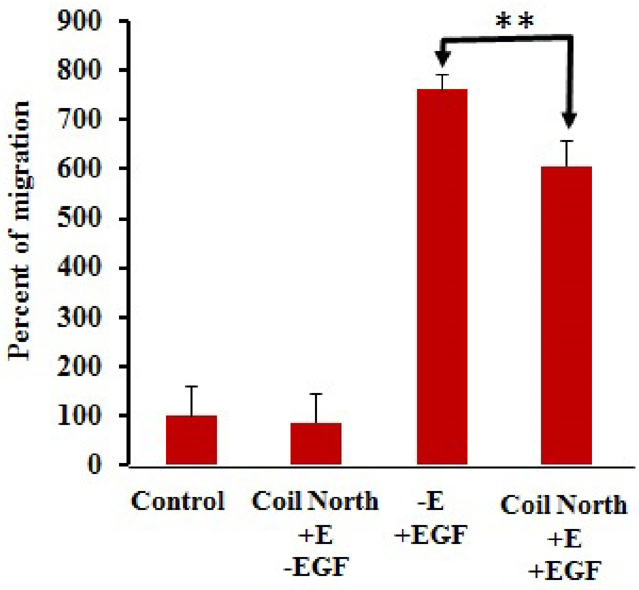

Summary of experimental results of migration of MCF-10A cells in our modified transmembrane assay showing the effects of induced electric fields with and without the growth factor EGF. These cell migration experiments were performed over a period of 16 hours on MCF-10A cells. These experiments were performed on the same apparatus and under the same conditions as in **Fig. 4**. **Column 1:** Control without induced E fields or EGF in our modified transmembrane assay (N=2). **Column 2:** Effect of induced E fields on MCF-10 cells in the absence of EGF on the "North" side of our modified transmembrane assay (N=2). **Column 3:** Control in our modified transmembrane assay with a Transwell insert, without induced E fields but in the presence of growth factor EGF in the lower chamber (N=4). **Column 4:** Migration is hindered on the "North" side of the coil in the presence of both EGF and induced electric fields (Column 3 versus Column 4: p=0.002), where for the majority (60%) of the 10 μs period, the induced E field is in the direction of migration (i.e. directed downward) (N=4).

Supplementary References

(R1) Smythe, W. R., *Static and Dynamic Electricity* (3rd ed.). New York: McGraw-Hill, 1968.

(R2) Abramowitz, M., Stegun, I.A. *Handbook of Mathematical Functions With Formulas, Graphs, and Mathematical Tables*, 10th Printing, National Bureau of Standards, Applied Mathematics Series, U.S. Government Printing Office, Washington, D.C., 1964.

(R3) McFerran, J. L., *An Electromagnetic Method of Cancer Detection*, Ph.D. dissertation, The Ohio State University, 2009.

(R4) A. Gilat, and V. V. Subramaniam, *Numerical Methods for Engineers and Scientists: An Introduction with Applications Using MATLAB,* John Wiley & Sons Inc., New York, 2014 (3rd Ed.).
